# Institute of Medicine Recommendations on the Rate of Gestational Weight Gain and Perinatal Outcomes in Rural Bangladesh

**DOI:** 10.3390/ijerph18126519

**Published:** 2021-06-17

**Authors:** S. M. Tafsir Hasan, Md Alfazal Khan, Tahmeed Ahmed

**Affiliations:** 1 Nutrition and Clinical Services Division, icddr,b, Dhaka 1212, Bangladesh; fazal@icddrb.org (M.A.K.); tahmeed@icddrb.org (T.A.); 2 Health System and Population Studies Division, icddr,b, Dhaka 1212, Bangladesh; 3 Office of the Executive Director, icddr,b, Dhaka 1212, Bangladesh

**Keywords:** gestational weight gain (GWG), rate of weight gain in the second and third trimester, pregnancy, Institute of Medicine (IOM), small for gestational age (SGA), large for gestational age (LGA), preterm birth, cesarean delivery, neonatal death

## Abstract

Although validated in other parts of the world, the suitability of the U.S. Institute of Medicine (IOM) 2009 recommendations on gestational weight gain (GWG) for Bangladeshi women remains to be examined. We evaluated the association between the weekly rate of weight gain during the second and third trimester of pregnancy, categorized according to IOM recommendations, and adverse perinatal outcomes among 1569 pregnant women with singleton live births in rural Matlab, Bangladesh. Gaining weight at rates below the IOM recommendations was associated with higher odds of preterm birth (adjusted odds ratio (AOR) = 2.0, 95% CI: 1.1–3.6), low birth weight (AOR = 1.4, 95% CI: 1.03–2.0), small-for-gestational-age newborns (AOR = 1.3, 95% CI: 1.04–1.7), and poor neonatal outcome (severe neonatal morbidity or death, AOR = 2.4, 95% CI: 1.03–5.6). A GWG rate above the recommendations was associated with higher odds of cesarean delivery (AOR = 1.7, 95% CI: 1.1–2.6), preterm birth (AOR = 2.2, 95% CI: 1.1–4.4), large-for-gestational-age newborns (AOR = 5.9, 95% CI: 1.5–23.1), and poor neonatal outcome (AOR = 2.7, 95% CI: 1.04–7.0). Our results suggest that the IOM 2009 recommendations on GWG rate during the second and third trimester may be suitable for guiding rural Bangladeshi women in the prenatal period, although the women should aim for rates near the lower bound of the range.

## 1. Introduction

Inappropriate gestational weight gain (GWG) is a widespread problem associated with adverse perinatal outcomes [1,2]. Low GWG is associated with an elevated risk of bearing low-birth-weight (LBW) and small-for-gestational-age (SGA) infants, while excessive GWG has been linked to an increased risk of childbirth by cesarean section, fetal macrosomia, and delivering large-for-gestational-age (LGA) newborns. Both low and excessive GWGs have been found to increase the risk of preterm birth and neonatal morbidity and mortality [1,3,4,5,6,7,8,9].

In 2009, the U.S. Institute of Medicine (IOM), nowadays known as the National Academy of Medicine, provided revised recommendations for total GWG as well as rates of weight gain during the second and third trimester of pregnancy. The recommendations depend on the prepregnancy body mass index (BMI). The recommended total GWGs for underweight, normal-weight, overweight, and obese women are 12.5–18, 11.5–16, 7–11.5, and 5–9 kg, respectively. The IOM also proposed that women should gain weight during the second and third trimester at the rate of 0.44–0.58, 0.35–0.50, 0.23–0.33, and 0.17–0.27 kg/week, respectively, for the same BMI stratification [9].

Our earlier study [10] evaluated the risk of intrauterine growth restriction (IUGR) related to low third-trimester weight gain in rural Bangladeshi women, using local standards of GWG first proposed in 2004 [11]. According to these standards, Bangladeshi women, irrespective of the prepregnancy BMI, are expected to put on no less than 9 kg of weight during the entire gestational period and greater than 4 kg in the third trimester [11]. We found that 56.3% of women gained lower than the recommendation in the third trimester, and low third-trimester GWG was associated with higher odds of IUGR [10].

Although the local standards were useful to gain some crucial insight into GWG status in Bangladesh, their wide application and potential integration into policy are constrained by several limitations. The local standards do not depend on prepregnancy BMI [10,11], whereas extensive evidence suggests that the range of GWG that supports optimal pregnancy outcomes varies according to prepregnancy BMI [9,12,13]. Second, these standards do not consider the possibility of excess weight gain and, thus, lack an upper limit—a major shortcoming in the wake of the obesity pandemic [9,12,13]. Third, goal setting for a recommended range of weekly rate of weight gain, which the local standards are lacking in [10,11], allows regular monitoring of women’s progress toward the goal and intervention at the earliest in the event of any deviations [9]. IOM guidelines, especially the recommendations for the rate of weight gain during the second and third trimester of pregnancy [9], are free of these limitations and can be a handy tool for guiding Bangladeshi women during the prenatal period for optimal perinatal outcomes as well as for real-time monitoring and evaluation of GWG status.

Although IOM recommendations were originally intended for use among American women [9], a recent meta-analysis [1] showed that these recommendations might also be appropriate for women living in western Europe and East Asia. Likewise, IOM recommendations may also be valid for Bangladeshi women. Unfortunately, due to lack of data, the review was unable to comment on the applicability of IOM guidelines to women in South Asia, including Bangladesh [1]. Comprehensive investigations into the suitability of IOM recommendations for Bangladeshi women are long overdue. The present study assessed whether gaining weight during the second and third trimester of pregnancy at rates outside the IOM 2009 recommended ranges was associated with adverse maternal, fetal, and neonatal outcomes among women in rural Bangladesh and, thus, examined the suitability of the recommendations in this population.

## 2. Materials and Methods

### 2.1. Study Design and Setting

This retrospective cohort study was conducted in Matlab, a rural area lying 55 km southeast of Dhaka, the capital of Bangladesh. The icddr,b (formerly the International Centre for Diarrhoeal Disease Research, Bangladesh (ICDDR,B)) runs a Health and Demographic Surveillance System (HDSS) in Matlab, covering a population of 230,000 [14]. Information on vital events, including births and deaths, and sociodemographic data are routinely collected. The icddr,b has a large central health facility in Matlab, namely Matlab Hospital, which offers free-of-cost care to women of childbearing age and children under the age of five residing in the icddr,b service area of HDSS. Maternal care services provided are routine antenatal care (ANC), normal vaginal delivery, dilation and curettage, and postnatal care. Although there are government-run primary healthcare facilities, women in this area preferentially seek ANC and maternity services from the icddr,b facilities. On average, 60–70% of pregnant women from the area are admitted to Matlab Hospital for delivery. However, many women end up giving birth at private clinics in the catchment area, mainly because Matlab Hospital does not perform cesarean deliveries. The details of the study setting have been reported previously [15].

### 2.2. Study Population and Data Source

**Figure 1:**
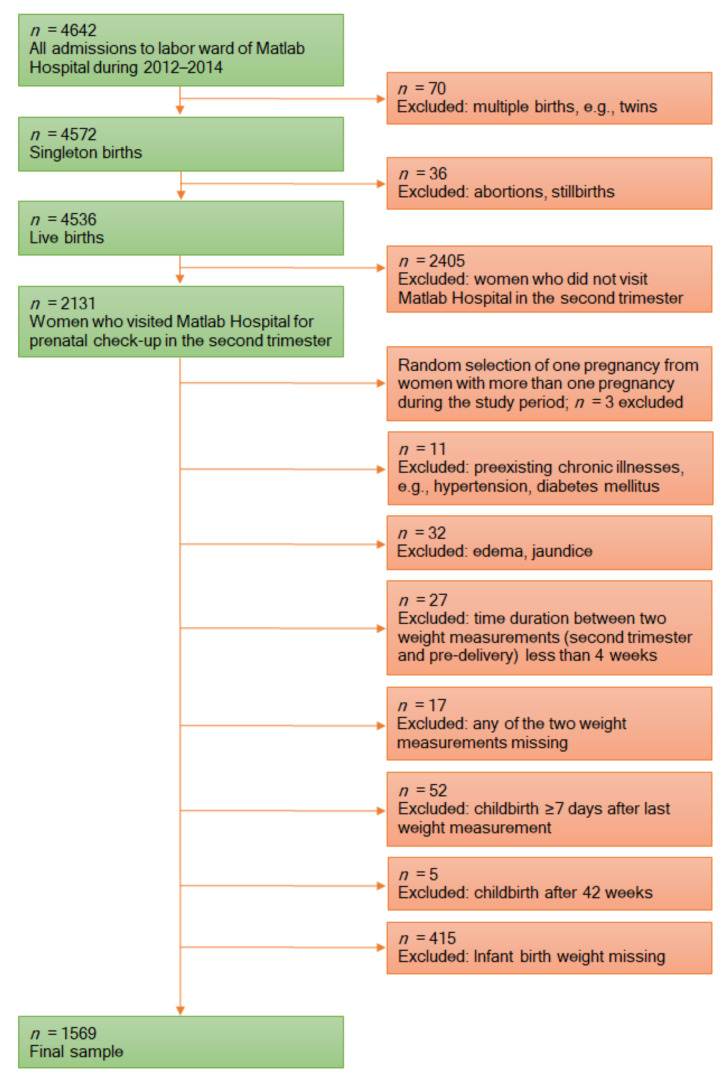
Flow diagram of the selection of the study population.

This study used data from the Pregnancy Weight Gain study, which evaluated the status of third-trimester GWG and its correlates among pregnant women admitted to the labor ward at Matlab Hospital in 2012–2014. The analysis included only those who also sought routine ANC from the same facility in the second trimester (23–29 weeks) of pregnancy. At Matlab Hospital, trained nurses and midwives took all anthropometric measurements following standard techniques. At the first prenatal visit, gestational age was estimated based on the reported date of the last menstrual period (LMP) and pregnancy ultrasound. The Pregnancy Weight Gain study retrieved all the necessary data from the electronic databases of Matlab Hospital and HDSS. The details of the study methodology have been reported elsewhere [16]. From the abovementioned study, data were available for 2131 women who gave live birth to singleton infants. In the present study, a complete case analysis was limited to 1569 mother–infant pairs. Figure 1 illustrates the selection of the study population for this analysis.

### 2.3. Prepregnancy BMI

Since most women in rural communities of resource-poor countries gain none to minimal weight in the first trimester (up until 16 weeks of pregnancy), weight recorded during this period can be used as a proxy for prepregnancy weight [9,17,18]. However, first-trimester weight was available for a subset of the study sample (*n* = 419). For the rest, first-trimester weight was estimated from an equation developed based on the subsample data using multiple linear regression modeling. After testing all covariates and nonlinearities, only second-trimester weight, gestational age at the time of second-trimester weight measurement, height, parity, and conception in winter were independently and significantly associated with first-trimester weight. The model was highly significant (*p* < 0.001), and the R-squared was 0.89. There was a strong correlation between actual and predicted first trimester weight (r = 0.94, *p* < 0.001). The final model was as follows:

First Trimester Weight (kg) = 24.28 + 0.95 × Second Trimester Weight (kg) − 0.48 × Gestational Age at Second Trimester Weight Measurement (weeks) − 0.09 × Height (cm) + 0.34 × Parity + 0.66 × Conception in Winter(1)

First-trimester BMI was calculated by dividing the first-trimester weight (actual or estimated) in kilogram by height in meter squared. In this study, first trimester BMI was used as a proxy for prepregnancy BMI. BMI was categorized according to the World Health Organization (WHO) definitions [19], as was followed for IOM recommendations [9].

### 2.4. Rate of Weight Gain during the Second and Third Trimester

The individual weekly rate of weight gain (kg/week) during the second and third trimester was calculated as follows:

Rate of Weight Gain (kg/week) = [Predelivery Weight (kg) − Second Trimester Weight (kg)] ÷ [Gestational Age at Predelivery Weight Measurement (weeks) − Gestational Age at Second Trimester Weight Measurement (weeks)](2)

The rate of weight gain was categorized as inadequate, optimal, or excessive if it was below, within, or above the IOM 2009 recommendations, respectively, for a specific prepregnancy BMI category [9].

### 2.5. Outcomes

Cesarean delivery, LBW, SGA, macrosomia, LGA, preterm birth, and poor neonatal outcome were the outcome variables in this study.

SGA, appropriate for gestational age (AGA), and LGA were defined as a birth weight less than the 10th percentile, within 10–90 percentiles, and above the 90th percentile, respectively, for specific sex and gestational age at birth [20,21] according to the international newborn standards from the INTERGROWTH-21st project [22]. LBW, normal birth weight (NBW), and macrosomia were defined as a birth weight below 2500 g, within 2500–4000 g, and equal to or above 4000 g, respectively, regardless of the sex of the newborn and the gestational age at birth [23,24].

Preterm birth was defined as the delivery of infants before 37 completed weeks of gestation [9]. Poor neonatal outcome was defined as the occurrence of severe neonatal morbidity or neonatal death. Neonatal death was defined as the death of an infant in the first 28 days after live birth [25]. Severe neonatal morbidity included birth asphyxia (5 min Apgar score < 7) [26,27], respiratory distress syndrome, neonatal sepsis, and seizures [7,28].

### 2.6. Statistical Analysis

We presented the characteristics of mothers and infants using percentage and median (interquartile range (IQR)), as appropriate. Exploratory restricted cubic spline (RCS) logistic regression was used to investigate any potential nonlinear relationship between the rate of weight gain during the second and third trimester of pregnancy and the adverse perinatal outcomes. We used four knots located at the 5th, 35th, 65th, and 95th percentiles of the distribution of the rate of GWG [6]. Predicted probabilities of each of the outcomes were obtained from the RCS logistic regression models and plotted against the range of the rate of GWG.

To assess the association of inadequate and excessive rate of weight gain, according to the IOM guidelines, with each of the binary outcomes, simple and multivariable binary logistic regression models were fitted. Conventional logistic regression was used for the following outcomes: cesarean delivery, preterm birth, LBW, and SGA. Firth logistic regression was used for macrosomia, LGA, and poor neonatal outcome as the prevalence of these outcomes was relatively low. Firth logistic regression provides more reliable estimates in instances of small sample size, complete separation, and rare events using penalized maximum likelihood estimation [29]. We expressed the strength of association as odds ratio (OR) and adjusted odds ratio (AOR) with 95% confidence interval (95% CI) using the optimal rate of weight gain as the referent. The discriminative performance (the ability to discriminate between those with and those without an outcome) of the covariate-adjusted logit models of IOM categories of rate of GWG for the detection of adverse perinatal outcomes was assessed based on the area under the receiver operating characteristic (AUROC) curve [30].

The sample was restricted to SGA and AGA infants for SGA analyses and LGA and AGA infants for LGA analyses. Likewise, we kept the sample limited to LBW and NBW infants for LBW analyses and infants with macrosomia and NBW for macrosomia analyses. The sample was restricted to the cases of primary cesarean delivery when assessing the association of inappropriate rate of weight gain with cesarean delivery. We could not identify the cesarean sections performed on maternal request or without proper clinical indications. However, existing data from Matlab indicate that clinically nonindicated cesarean sections are especially common among relatively highly educated women [31]. Hence, we built two separate sets of models for primary cesarean delivery: one for the women who received less than ten years of education and another for the women who completed at least ten years of formal education. Completing secondary education (10 years of schooling) was considered higher education for women based on previous studies in this population [10,16] and the Government’s emphasis on girls’ completion of secondary education to leverage the better-educated female labor force to boost the ongoing socioeconomic development in Bangladesh [32,33].

All the multivariable models included the following covariates (maternal characteristics) of a priori interest: age (≤19 years, 20–34 years, ≥35 years), height (≤145 cm, >145 cm), prepregnancy BMI (underweight, normal weight, overweight/obese), parity (nulliparous, 1 prior birth, 2 prior births, 3 or more prior births), level of education (≤5 years, 6–9 years, ≥10 years), and wealth quintile. The obese category of prepregnancy BMI was merged with the overweight category because obese women were very few. Wealth quintile, an indicator of household-level wealth consistent with expenditure and income measures, was generated by HDSS based on household asset data using principal component analysis [34]. The multivariable model examining the association of inappropriate weight gain with “poor neonatal outcome” included infant sex in addition to the abovementioned covariates. The selection of covariates (and their categorization) was guided by previous studies on risk factors of the study outcomes and studies investigating their associations with GWG [7,9,10,16,35,36,37]. We also examined the role of prepregnancy BMI as an effect modifier but found no evidence for a statistically significant interaction effect in our data.

All statistical tests were two-sided, and statistical significance was evaluated at *p* < 0.05. Data analysis was done in STATA v15.1 (StataCorp, College Station, TX, USA). Birth weight percentiles were calculated using the INTERGROWTH-21st “Neonatal Size Calculator for newborn infants between 24 + 0 and 42 + 6 weeks’ gestation”.

## 3. Results

Of the 1569 women in the study, 20.3% were adolescents, and 6.4% were aged 35 years or above. Fifteen percent of women had a height less than or equal to 145 cm, and 42.1% were nulliparous. Of the women, 20.7% completed at least ten years of formal education (Table 1).

**Figure 2:**
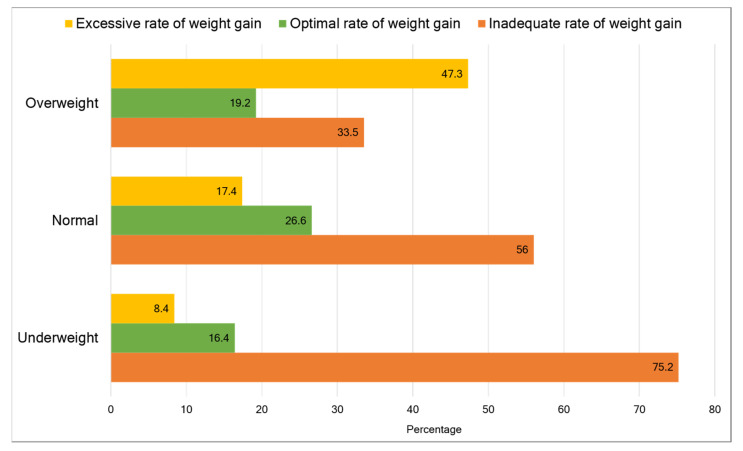
Percent distribution of categories of rate of gestational weight gain (according to the Institute of Medicine 2009 guidelines) during the second and third trimester among underweight, normal-weight and overweight women. The overweight category includes overweight and obese women.

In our study, 15.2% of women were underweight, and 13% were overweight (or obese) at the beginning of pregnancy. Of the women, 56% gained weight at a rate below the IOM 2009 recommendations, 24.1% gained within the recommended rates, and 19.9% gained at a rate above the recommendations during the second and third trimester of pregnancy (Table 1). Figure 2 shows that inadequate rate of GWG was more frequent among underweight women (75.2%, 56.0%, and 33.5% in underweight, normal-weight, and overweight women, respectively). In contrast, excessive rate of GWG was more common among overweight women (47.3%, 17.4%, and 8.4% in overweight, normal-weight, and underweight women, respectively).

**Figure 3:**
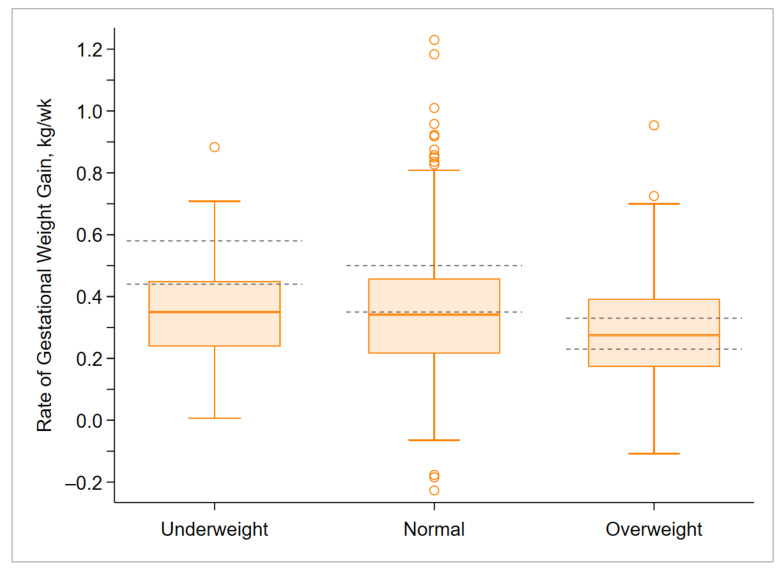
Rate of gestational weight gain during the second and third trimester among underweight (*n* = 77), normal-weight (*n* = 445), and overweight (*n* = 83) women who had optimal perinatal outcomes (no adverse outcomes). The overweight category includes overweight and obese women. Boxes indicate medians and interquartile ranges. Error bars indicate ranges. Circles indicate outliers. Gray dashed lines indicate the Institute of Medicine recommended ranges.

The median (IQR) rate of GWG among underweight, normal-weight, and overweight women who had optimal perinatal outcomes (no adverse outcomes) was 0.35 (0.24, 0.45), 0.34 (0.22, 0.46), and 0.28 (0.17, 0.39) kg/week, respectively. Although the IQR of the rate of GWG overlapped with the IOM-recommended ranges for all three prepregnancy BMI categories, a large portion of the distribution of the rate of GWG was below the recommendations in case of underweight and normal-weight women (Figure 3).

Overall, 24.3% of women had a cesarean delivery. The prevalence of cesarean delivery was 21.3% among women who received less than ten years of education, but it was 35.8% among those who completed at least ten years of schooling (results not shown in the tables). In our sample, 14 (0.9%) individuals had a repeat cesarean section. Among the infants, 18.1% were LBW, and 38.4% were SGA at birth. Macrosomia was found in 1.4% of infants, and 2.1% were LGA at birth. Of the infants, 6.8% had a preterm birth. Severe neonatal morbidity occurred in 2.7% of infants, and 19 (1.2%) infants died within 28 days after live birth (Table 1).

**Figure 4:**
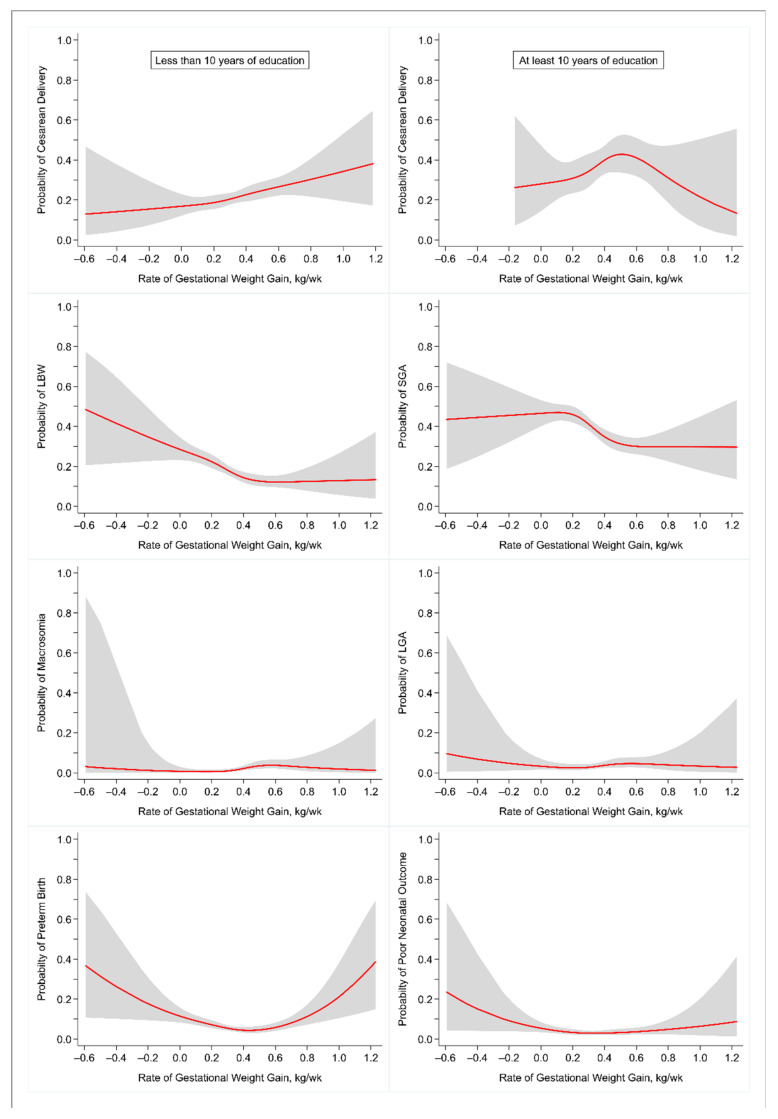
Predicted probability (95% CI) of adverse perinatal outcomes for rate of gestational weight gain during the second and third trimester. Abbreviations: CI, confidence interval; LBW, low birth weight; SGA, small for gestational age; LGA, large for gestational age.

Figure 4 shows the shape of associations between the continuous rate of GWG during the second and third trimester and the predicted probability of adverse perinatal outcomes obtained from the RCS logistic regression models. The plots indicate that more than one adverse perinatal outcome may have a nonlinear (U-shaped) association with the rate of GWG; however, only preterm birth showed a statistically significant nonlinearity (*p* < 0.001).

Table 2 and Table 3 show the bivariable and multivariable association of inadequate and excessive rate of gestational weight gain with adverse maternal, fetal, and neonatal outcomes. In adjusted models, an inadequate rate of GWG during the second and third trimester was associated with higher odds of preterm birth (AOR = 2.0, 95% CI: 1.1, 3.6), LBW (AOR = 1.4, 95% CI: 1.03, 2.0), SGA (AOR = 1.3, 95% CI: 1.04, 1.7), and poor neonatal outcome (AOR = 2.4, 95% CI: 1.03, 5.6). No statistically significant association was found between gaining weight at a rate less than the IOM recommendation and cesarean delivery, macrosomia, or LGA (Table 2).

On the other hand, a GWG rate above the IOM recommendations was associated with higher odds of preterm birth (AOR = 2.2, 95% CI: 1.1, 4.4), LGA (AOR = 5.9, 95% CI: 1.5, 23.1), and poor neonatal outcome (AOR = 2.7, 95% CI: 1.04, 7.0). Macrosomia was marginally significant (AOR = 3.3, 95% CI: 1.0, 10.9; *p* = 0.051). Excessive rate of GWG was associated with higher odds of cesarean delivery in women who received less than ten years of education (AOR = 1.7, 95% CI: 1.1, 2.6); however, no statistically significant association was found among those who completed at least ten years of education. Gaining weight at a rate higher than the IOM recommendation showed no statistically significant association with LBW or SGA (Table 3).

**Figure 5:**
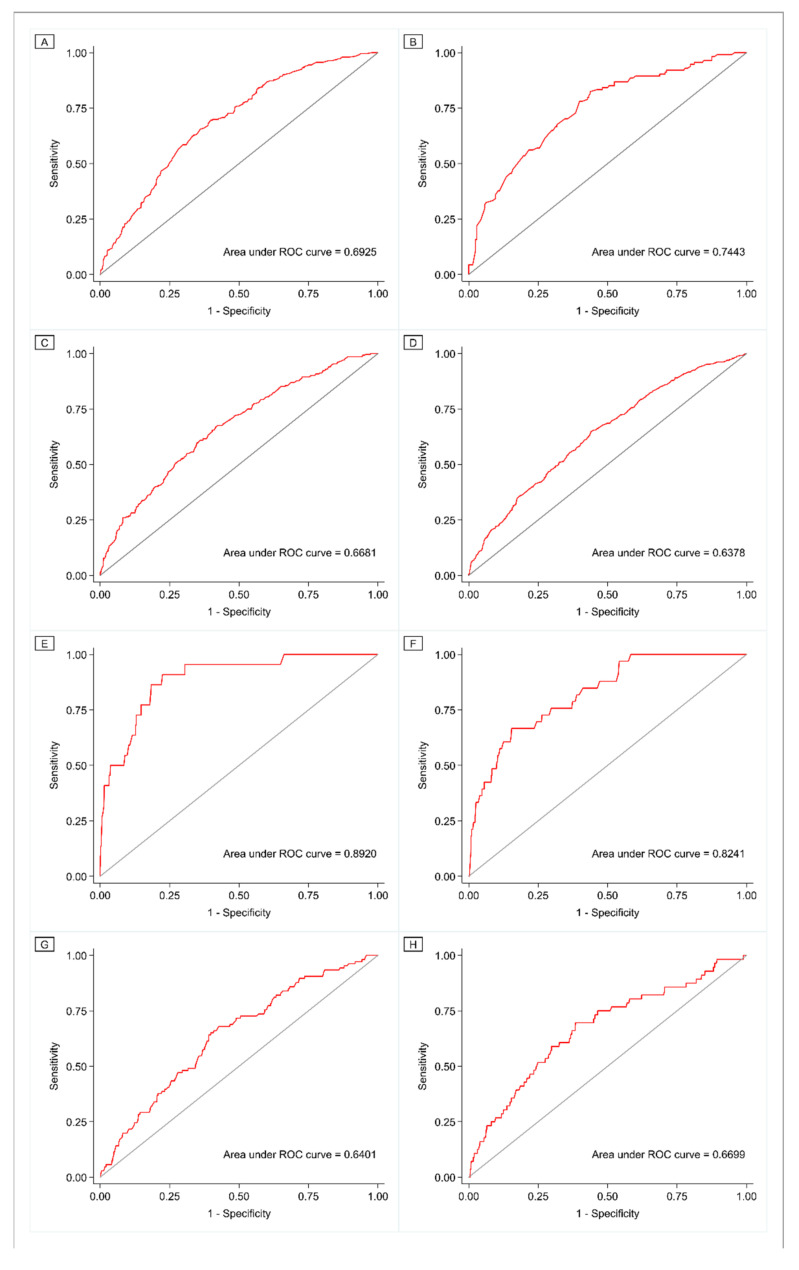
Receiver operating characteristic curves of the covariate-adjusted logit models of IOM categories of rate of gestational weight gain for the detection of adverse perinatal outcomes ((**A**) Cesarean delivery among women who received less than ten years of education, (**B**) cesarean delivery among women who completed at least ten years of education, (**C**) LBW, (**D**) SGA, (**E**) macrosomia, (**F**) LGA, (**G**) preterm birth, (**H**) poor neonatal outcome). Abbreviations: ROC, receiver operating characteristic; LBW, low birth weight; SGA, small for gestational age; LGA, large for gestational age.

The covariate-adjusted models of IOM categories of rate of GWG showed modest to strong ability to distinguish between those with and those without adverse perinatal outcomes (AUROC range: 0.64–0.89) (Figure 5).

## 4. Discussion

We studied the association of several perinatal outcomes with IOM recommendations on the rate of GWG during the second and third trimester of pregnancy. To our knowledge, ours is the first study to examine the suitability of IOM guidelines on GWG for Bangladeshi women, especially taking into consideration varied maternal, fetal, and neonatal outcomes. The results showed that rates of GWG during the second and third trimester outside the ranges recommended in the IOM 2009 guidelines were associated with adverse perinatal outcomes, independent of prepregnancy BMI and other maternal and neonatal characteristics.

In our sample, women who gained weight at rates below the IOM-recommended ranges during the second and third trimester of pregnancy had a higher risk of delivering LBW and SGA infants than those whose weight gain rates were within the ranges. On the other hand, women who gained weight at rates above the IOM-recommended ranges were more likely to have macrosomic and LGA babies at birth compared to those whose weight gain rates were within the ranges. Such associations are biologically plausible because rapid fetal growth and fat accretion occur during the late second and third trimester of pregnancy, and fetal weight is a component of GWG [9,38,39]. However, we did not find any significant association between an excessive rate of GWG and SGA or between inadequate rate of GWG and LGA in the adjusted models. In a recent study among nulliparous American women with singleton pregnancies, Dude et al. showed that GWG rates in the second and third trimester below and above IOM recommendations were associated with higher odds of SGA (AOR = 1.37, 95% CI: 1.08, 1.73) and LGA infants at birth (AOR = 1.34, 95% CI: 1.01, 1.79), respectively. Although the study did not find any link between low weight gain and LGA, unlike us, they found that excessive rate of weight gain significantly reduced the risk of SGA babies [40]. A retrospective chart analysis of data from 29,861 women in 25 U.S. hospitals found higher odds of macrosomia among women with rates of GWG above IOM recommendations (AOR = 2.66, 95% CI: 2.03, 3.48) but did not find any association with weight gain below the recommendations [5]. In another recent study in two rural sub-districts in northwest Bangladesh, compared to women with inadequate GWG during the second and third trimester, women who gained weight at a rate within the IOM guidelines had a lower risk of delivering LBW and SGA infants [41].

In our study, the rate of weight gain showed a U-shaped relationship with preterm birth; both inadequate and excessive rates of GWG during the second and third trimester were associated with a heightened risk of preterm delivery. There are no clear biologic mechanisms for the intriguing association between the rate of weight gain and preterm birth. However, it has been proposed that maternal undernutrition may increase the risk of preterm birth by suppressing immune functions or increasing oxidative stress [9]. In a recent study, Huang et al. also found this U-shaped relationship among Chinese women. After adjustment for potential confounders, both low (AOR = 1.41, 95% CI: 1.07, 1.85) and high (AOR = 1.37, 95% CI: 1.11, 1.68) GWG rates, according to IOM 2009 recommendations, were associated with higher odds of preterm birth [42].

Our results showed that similar to preterm birth, poor neonatal outcome had a U-shaped relationship with the rate of GWG. Rates of weight gain below and above the IOM recommendations were associated with an elevated risk of poor neonatal outcome. In this study, poor neonatal outcomes ranged from severe neonatal illnesses such as birth asphyxia, respiratory distress syndrome, sepsis, and seizures to death. A recent population-based retrospective cohort study among pregnant women with singleton births in the USA, between 2004 and 2013, showed that GWG below the IOM recommendations was associated with higher odds of perinatal death and serious neonatal morbidities, including respiratory distress syndrome, neonatal sepsis, severe birth trauma, and neonatal seizures. GWG above the recommendations also increased the risk, however, only in underweight and normal-weight women [7].

The present study also found that an excessive rate of GWG was associated with higher odds of cesarean delivery among women who received less than ten years of education. This is consistent with recent literature [5,6,43]; cesarean delivery is a common consequence of excessive GWG. However, we did not observe this association in women who completed at least ten years of education. In Bangladesh, the rate of clinically non-indicated cesarean deliveries is increasing [31,44,45]. Surprisingly, this scenario is especially prevalent among highly educated women who can make an informed decision about the mode of delivery [31], which is in stark contrast to the picture observed in high-income countries, where higher education and economic status have been found to be protective against cesarean delivery [46]. In resource-poor settings, the conduct of cesarean delivery in the absence of any clinical indications could be a means of defensive obstetric practice [45,47].

Consistent with findings from studies in countries that adopted the IOM 2009 guidelines, gaining weight within the IOM-recommended ranges during the second and third trimester of pregnancy seemed to help women in rural Matlab achieve optimal perinatal outcomes. The results also support the idea that regular monitoring of the rate of GWG, early detection of any deviations, and taking prompt action for correction may be crucial to avert adverse perinatal outcomes [43]. Poor dietary diversity, lack of awareness of the necessity of adequate GWG and a balanced diet in pregnancy, cultural norms such as women’s eating last at a meal resulting in the smallest share, lack of involvement in household decision-making, and household food insecurity are thought to be among the key contributors to poor GWGs in rural Bangladesh [10,11,48]. Attending frequent and focused prenatal counseling sessions, with an emphasis on a balanced diet and regular weight monitoring, can help these women achieve optimal GWG [9,49]. For a positive pregnancy experience, prenatal care, including counseling, should be initiated at the earliest [49]. Targeted supplementations and social safety net programs can be provisioned for women from food-insecure households.

Although our results showed that rates of GWG outside the IOM-recommended ranges were associated with adverse perinatal outcomes, the discriminative performance of the covariate-adjusted GWG models ranged from modest to strong. This may be a general case with GWG recommendations rather than a population-specific finding as the same also has been observed in a recent individual participant-level meta-analysis using data from 25 cohort studies from North America and Europe [30]. Another thing to consider is the rates of GWG among women with optimal perinatal outcomes in our sample were generally found to be at the lower end of the ranges recommended in the IOM 2009 guidelines. Interestingly, this is consistent with the IOM 1990 recommendation that “short women (<157 cm) should strive for gains at the lower end of the range [39]”. Although not specified in the revised 2009 guidelines, we believe the 1990 recommendation for short women may still apply to generally short-statured Bangladeshi women. Another surprising finding was that the average rate of GWG among underweight women with optimal outcomes was comparable to that among normal-weight women with optimal outcomes. This is in stark contrast to the available evidence and IOM guidelines [9], which recommend a higher rate of weight gain for underweight women. This result might simply indicate a lack of statistical power due to the small sample size of underweight women rather than having any biologically plausible explanation. Overall, our results suggest that obstetricians, nurses, and health workers may follow the recommendations on the rate of GWG stipulated in the IOM 2009 guidelines to set weekly weight gain goals for pregnant women in Bangladesh, monitor the progress during the gestational period, and counsel accordingly. However, Bangladeshi women, especially those who are short-statured, should set goals for the weekly rate of GWG near the lower end of the recommended range according to the prepregnancy (first trimester) BMI.

In our study, although both inadequate and excessive rates of GWG were found to be associated with adverse perinatal outcomes, the proportion of women experiencing suboptimal GWG rates during the second and third trimester was substantially higher than the proportion of those who gained at rates above the IOM recommendations (56% vs. 19.9%). A recent study that followed pregnant women in two rural sub-districts in northwest Bangladesh from 9–16 weeks to 36 weeks of gestation, showed that 74% of women were gaining weight at rates less than the IOM recommendations [41]. The National Low-Birth-Weight Survey 2015 estimated that 68% of the women in Bangladesh gained less than 7 kg during the entire pregnancy period [48]. It is evident that maternal undernutrition and inadequate GWGs still prevail in Bangladesh despite the emergence of the obesity epidemic. We also found that an inadequate rate of weight gain was most frequent among underweight women, and excessive weight gain was common among overweight women. These findings are in line with that of other studies in low- as well as high-income countries [6,7,8,9,41] and highlight the importance of starting pregnancy with a normal body weight and preconception counseling.

The study has several limitations. The study used routinely collected service and surveillance data retrieved from electronic databases. Routine data are well known to have errors and biases, including data linkage problems and misclassification bias [50,51]. Many women ended up being excluded due to lack of data and for other reasons; however, both the women included in the analysis as well as those excluded were from the same homogeneous population in Matlab. We used first-trimester BMI as a proxy measure for prepregnancy BMI because information on prepregnancy weight was not available in the database. Furthermore, the first-trimester weight was available for only a subset of the population; hence, we had to estimate the first-trimester weight for women who were first weighed after 16 weeks of gestation. However, similar methods have been used successfully to estimate prepregnancy BMI in populations lacking accurate knowledge of prepregnancy weight [41,52]. Our data showed a strong correlation between the actual and the predicted weight in the first trimester. Contemporary nationally representative data also showed a somewhat similar distribution of BMI categories among women of reproductive age [44]. This study estimated the rate of weight gain during the second and third trimester for each woman, assuming that women gained weight at a somewhat steady pace from the time of prenatal checkup in the second trimester up until delivery. Although supported by strong evidence [9], the assumption may not apply to women who experienced variable weight gain rates throughout the pregnancy. Furthermore, our analysis could not separate the role of weight gain rate in the second trimester from that in the third trimester due to lack of data. Additionally, some residual confounding might be present because the study lacked data on dietary intake and diversity, physical activity, nausea/vomiting, and psychosocial factors. Studies in other parts of Bangladesh are required to replicate these findings to confirm the nationwide generalizability.

## 5. Conclusions

This study demonstrated that, among women in rural Matlab, gaining weight at a rate below or above the IOM recommendations adversely affected maternal, fetal, and neonatal outcomes. Our findings suggest that the IOM 2009 recommendations on the rate of GWG during the second and third trimester may be suitable for guiding rural Bangladeshi women in the prenatal period. However, Bangladeshi women, especially those who are short-statured, ought to aim for rates near the lower bound of the range. To promote optimal perinatal outcomes, prenatal care may emphasize nutrition counseling and regular weight monitoring following IOM guidelines.

## Figures and Tables

**Table 1:** Characteristics of the study population.

Characteristics	*n* (%)
Maternal characteristics	
Age	
≤19 years	319 (20.3)
20–34 years	1149 (73.2)
≥35 years	101 (6.4)
Height, ≤145 cm	235 (15.0)
Prepregnancy BMI	
Underweight (<18.5 kg/m^2^)	238 (15.2)
Normal (18.5–24.9 kg/m^2^)	1128 (71.9)
Overweight (25–29.9 kg/m^2^)	185 (11.8)
Obese (≥30 kg/m^2^)	18 (1.2)
Parity	
Nulliparous	660 (42.1)
1 previous birth	436 (27.8)
2 previous births	307 (19.6)
≥3 previous birth	166 (10.6)
Previous cesarean delivery	14 (0.9)
Education, years	
≤5	367 (23.4)
6–9	878 (56.0)
≥10	324 (20.7)
Wealth quintile	
Lowest	249 (15.9)
Second	269 (17.1)
Middle	303 (19.3)
Fourth	327 (20.8)
Highest	421 (26.8)
Rate of weight gain	
Inadequate	879 (56.0)
Optimal	378 (24.1)
Excessive	312 (19.9)
Preterm labor	106 (6.8)
Cesarean delivery	381 (24.3)
Offspring characteristics	
Sex, Female	773 (49.3)
LBW	284 (18.1)
SGA	602 (38.4)
Macrosomia	22 (1.4)
LGA	33 (2.1)
Severe neonatal morbidity	42 (2.7)
Neonatal death	19 (1.2)
Poor neonatal outcome	56 (3.6)

Abbreviations: BMI, body mass index; LBW, low birth weight; SGA, small for gestational age; LGA, large for gestational age.

**Table 2:** Association of inadequate rate of gestational weight gain during the second and third trimester with adverse maternal, fetal, and neonatal outcomes.

Adverse Outcome	OR (95% CI)	*p*	AOR (95% CI) ^1^	*p*
Maternal				
Cesarean delivery in women who received less than 10 years of education	0.9 (0.6, 1.3)	0.502	0.9 (0.6, 1.3)	0.452
Cesarean delivery in women who completed at least 10 years of education	0.6 (0.3, 1.1)	0.080	0.6 (0.3, 1.1)	0.105
Fetal				
LBW	1.6 (1.1, 2.2)	0.006	1.4 (1.03, 2.0)	0.034
SGA	1.5 (1.2, 1.9)	0.002	1.3 (1.04, 1.7)	0.023
Macrosomia	0.5 (0.1, 2.1)	0.314	0.6 (0.1, 2.5)	0.450
LGA	3.0 (0.8, 11.4)	0.116	3.2 (0.8, 12.5)	0.094
Preterm birth	2.1 (1.2, 3.7)	0.013	2.0 (1.1, 3.6)	0.017
Neonatal				
Poor neonatal outcome	2.5 (1.1, 5.8)	0.035	2.4 (1.03, 5.6)	0.043

^1^ AOR (95% CI) of cesarean delivery for inadequate rate of gestational weight gain was obtained from multivariable models that were adjusted for maternal age, height, prepregnancy BMI, parity, and wealth quintile. AORs (95% CI) of LBW, SGA, macrosomia, LGA, and preterm birth were obtained from multivariable models that were adjusted for maternal age, height, prepregnancy BMI, parity, education, and wealth quintile. AOR (95% CI) of poor neonatal outcome was obtained from a multivariable model that was adjusted for maternal age, height, prepregnancy BMI, parity, education, wealth quintile, and infant sex. Abbreviations: OR, odds ratio (unadjusted); AOR, adjusted odds ratio; CI, confidence interval; LBW, low birth weight; SGA, small for gestational age; LGA, large for gestational age; BMI, body mass index.

**Table 3:** Association of excessive rate of gestational weight gain during the second and third trimester with adverse maternal, fetal, and neonatal outcomes.

Adverse Outcome	OR (95% CI)	*p*	AOR (95% CI) ^1^	*p*
Maternal				
Cesarean delivery in women who received less than 10 years of education	1.8 (1.2, 2.8)	0.003	1.7 (1.1, 2.6)	0.023
Cesarean delivery in women who completed at least 10 years of education	1.3 (0.7, 2.4)	0.395	0.9 (0.5, 1.8)	0.753
Fetal				
LBW	0.6 (0.4, 1.02)	0.060	0.7 (0.4, 1.2)	0.183
SGA	0.7 (0.5, 0.96)	0.029	0.8 (0.5, 1.1)	0.110
Macrosomia	5.6 (1.8, 18.1)	0.004	3.3 (1.0, 10.9)	0.051
LGA	7.9 (2.1, 30.3)	0.002	5.9 (1.5, 23.1)	0.010
Preterm birth	1.8 (0.9, 3.6)	0.077	2.2 (1.1, 4.4)	0.023
Neonatal				
Poor neonatal outcome	2.8 (1.1, 7.1)	0.032	2.7 (1.04, 7.0)	0.041

^1^ AOR (95% CI) of cesarean delivery for excessive rate of gestational weight gain was obtained from multivariable models that were adjusted for maternal age, height, prepregnancy BMI, parity, and wealth quintile. AORs (95% CI) of LBW, SGA, macrosomia, LGA, and preterm birth were obtained from multivariable models that were adjusted for maternal age, height, prepregnancy BMI, parity, education, and wealth quintile. AOR (95% CI) of poor neonatal outcome was obtained from a multivariable model that was adjusted for maternal age, height, prepregnancy BMI, parity, education, wealth quintile, and infant sex. Abbreviations: OR, odds ratio (unadjusted); AOR, adjusted odds ratio; CI, confidence interval; LBW, low birth weight; SGA, small for gestational age; LGA, large for gestational age; BMI, body mass index.

## Data Availability

All aggregated data are provided within the paper. To protect the identification of participants derived from the composite of key study variables, some restrictions do apply to the primary data. These data can be made available from the Ethics Committees (RRC and ERC) of icddr,b for researchers who meet the criteria for access to confidential data. Please contact the Head of Research Administration at the icddr,b (Armana Ahmed; aahmed@icddrb.org) for data policies and queries.
